# Soil–Plant Characterization in Agrosilvopastoral System Established in a Fe-Mn Abandoned Mine After Long-Term Closure

**DOI:** 10.3390/plants14010060

**Published:** 2024-12-27

**Authors:** Erika S. Santos, Maria Manuela Abreu, Sabina Rossini-Oliva

**Affiliations:** 1 LEAF—Linking Landscape, Environment, Agriculture and Food Research Center, Associate Laboratory TERRA, Instituto Superior de Agronomia, Universidade de Lisboa, Tapada da Ajuda, 1349-017 Lisbon, Portugal; manuelaabreu@isa.ulisboa.pt; 2 Department of Plant Biology and Ecology, Universidad de Sevilla, Avda. Reina Mercedes S/N, 41080 Seville, Spain; sabina@us.es

**Keywords:** dryland pasture, herbaceous cover, holm oak, biogeochemical characterization, environmental risk

## Abstract

Small abandoned mining areas of Fe and Mn oxides located in the Portuguese sector of the Iberian Pyrite Belt (SW of Europe) have been converted into agrosilvopastoral systems with very few environmental management measures after their closure. Although at the landscape scale, no visible differences were observed between the former mining intervention areas and adjacent areas, it is essential to assess the state and environmental risk of the soil–plant system, especially in the herbaceous pastures grazed by domestic animals. This was carried out in the Ferragudo mining area, where an agrosilvopastoral system, composed of holm oak and dryland pasture, had been established after the closure of the mine at ≈45 years. The soils presented neutral pH and variable fertility degree. The pseudo-total soil concentrations of Cu, Mo, and Zn exceeded the Portuguese limit values established for agriculture use (>180 mg Cu/kg; >8.2 mg Mo/kg; 349 mg Zn/kg), but their soil available fractions were small (<8.4% of the pseudo-total concentrations). Trees and herbaceous plants showed good development, and the concentrations of the elements (except Mn) were considered normal or sufficient. For Mn, most of the plant samples exceeded phytotoxic Mn values, but no visual signs of phytotoxicity were observed. Only the concentrations of Fe and K in the shoots of some herbaceous samples exceeded the maximum tolerable levels for cattle and sheep, so the risk to animals can be considered small since other sources are present in animal feed. In general, this agrosilvopastoral system did not pose a significant environmental risk.

## 1. Introduction

Mining activities affect approximately 4 × 10^5^ km^2^ of land worldwide [1] and can leave a degraded area if environmental management issues are not considered during and after closure. Ancient mining activity in the Portuguese sector of the Iberian Pyrite Belt (PIPB, SW of Europe) has led to several environmental problems due to the existence of very few (if any) measures to manage and minimize environmental or human health risks [2,3]. In Portugal, until the XXth century, there was no legal obligation to rehabilitate land after the closure of mines. Thus, in some small/medium abandoned mines, the first post-closure activities were focused on the dispersion of mine wastes with/without mixing with natural soils. Some of these areas were reconverted to agriculture, pastoralism, forestry, or a combination of these (agrosilvopastoral systems) by the local population and the national entity responsible for environmental rehabilitation (Exmin), as well as left to natural plant colonization.

Independently of the high total concentrations of potentially hazardous elements in soils from PIPB mining areas, the available fraction is usually low, allowing the development of some plant species [2,4]. In fact, some abandoned mining areas (e.g., mining areas of Mn and Fe oxides) have been classified as having low/medium environmental risk [3,5], and no obvious landscape-scale differences have been observed between the former mining intervention area and adjacent areas.

Although the environmental recovery processes associated with plant development (natural attenuation and phytostabilization) are slow, especially in contaminated areas, these can contribute to improving soil quality [6,7] and minimizing land degradation. The improvement of soil quality by plants depends, for example, on the species, the strata type (herbaceous, shrubs, and trees), the type of root system, and its development. In addition, vegetation development can improve soil structure and fertility, promote biological activity, and increase water retention capacity [8].

The agrosilvopastoral system is a type of land use that combines pasture or fodder production with a woody component of shrubs and trees, with different livestock complementing each other in the same area [9,10]. This integrated system can contribute to several environmental benefits and ecosystem services, such as reducing soil degradation or even improving some soil properties and increasing C stocks, thus ensuring fodder availability at least during the rainy season [9,10,11]. Although the effect of seasonality is more pronounced in dryland systems, the combination of legume and grass species, perennial and annual herbaceous species, shrubs, and trees promotes complementarity, sustainability, and resilience of the system.

According to different characteristics of soils and intrinsic plants, plants growing in contaminated soils may uptake and accumulate different levels of potentially hazardous elements in edible parts [12,13]. This can lead to a potential risk to human and animal health when the plants are consumed; therefore, an assessment of the elemental composition of the plants is needed before making a decision on land use. In this sense, the bioaccumulation of elements in the soil–plant–animal system has been studied by some authors [14,15,16,17,18]. The aim of this study was to evaluate the general status and potential environmental risk of the soils and plants (herbaceous shoots of the dryland pasture and holm oak leaves) in an agrosilvopastoral system established in a Fe-Mn mine from Portugal after long-term closure (≈45 years ago).

## 2. Results and Discussion

**Figure 1:**
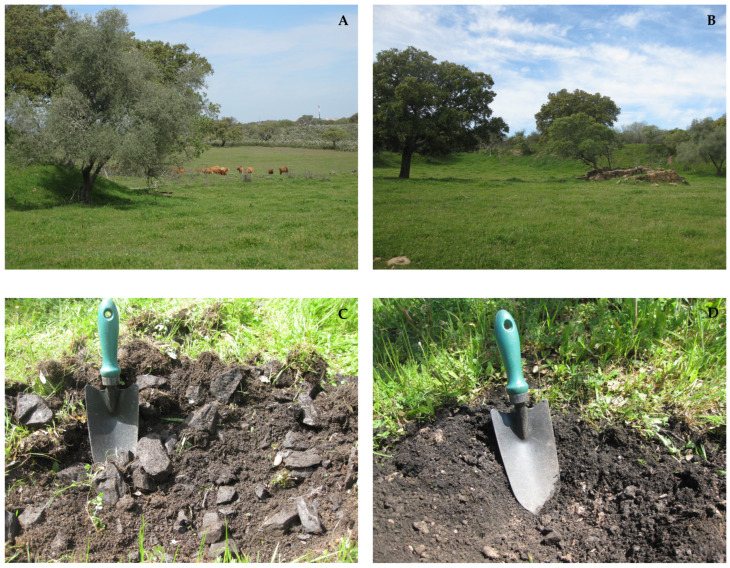
Agrosilvopastoral system established in Ferragudo mine area (**A**,**B**) and collection of soil samples (**C**,**D**).

This study presents a general evaluation of the quality of soils and plants growing in an agrosilvopastoral system established ≈45 years after the closure of the Ferragudo mine (Figure 1A,B). Soils from the holm oak area are under the influence of herbaceous and tree rhizosphere systems and canopy, while soils from the pasture area have only herbaceous roots.

Independently of the area sampled (designated as holm oak or herbaceous pasture) and depth, the soils had neutral pH values and a slightly acid reaction, indicated by the pH in KCl, with low electrical conductivity (Table 1) being considered non-saline soils. These parameter values coincide with very low total acidity (Table 1), corresponding to the concentration of Al and H^+^ in the exchangeable complex of the colloidal fraction. The concentrations of organic C and nutrients (Table 1) showed some variability. Thus, the concentrations of organic C and extractable P varied from very small (4.4 g C_org_/kg and <10 mg P/kg) to very high (>60 g C_org_/kg and >90 mg P/kg), while extractable K was mainly considered as high to very high [19,20]. Cation exchange capacity was considered between medium to high (10–40 cmol_c_/kg [15]) and was correlated to organic C amounts (r ≈ 0.8, *p* < 0.05). The exchangeable complex was dominated by Ca. In soils collected under holm oak trees, the concentrations of organic C, N total, and extractable K and P decreased significantly with depth (Table 1) because most of the roots are located between 10 and 20 cm depth, and most of the element uptake occurs.

The studied soils presented higher fertility, evaluated by the concentrations of organic C and NPK, than the Spolic Technosols collected in a similar mining area (Rosalgar) and the uncontaminated soils of the region, both naturally colonized by autochthonous vegetation [4,21]. This may indicate a direct positive effect of soil management, possibly with some fertilizer application to the whole surface and/or the agrosilvopastoral system itself. The improvement of soil fertility in the agrosilvopastoral systems due to the increase in nutrients and organic matter inputs has been reported [22]. The soils under the holm oak canopy did not show a similar richness in organic matter and nutrients as those outside the canopy (Table 1), which is not usually observed [10,23]. In semi-natural systems with holm oak (dehesa), the tree cover leads to an increase in soil fertility due to the accumulation of some fallen leaves and the reduced decomposition of organic matter in the shaded area under the tree crowns [10,11,23].

In general, there was no great heterogeneity in the chemical characteristics of the soils from holm oak and herbaceous pasture cover (Table 1), although values outside the main range were obtained for some samples (e.g., extractable K in a sample from herbaceous cover; extractable P in a superficial sample from holm oak; total N in a sample from holm oak at the second depth). Although a variation in soil particle size distribution associated with the irregular disposal of mine wastes (e.g., slags) was visually observed (Figure 1C,D), no heterogeneity in soil chemical characteristics was obtained. This small variation in the chemical characteristics can be related to the continuous soil management since the mine closure, which allowed the mixing of different mine materials (e.g., slags and other mine wastes, host rocks) with natural soil and the establishment of a chemical equilibrium over time.

Soils from both plant systems had concentrations of Cu, Mo, and Zn in the pseudo-total fraction (Table 2) that exceeded the reference values proposed by Portuguese legislation for shallow soils and agricultural use (180 mg Cu/kg; 6.9 mg Mo/kg; 340 mg Zn/kg [24]). Special attention should be paid to Mo, whose concentrations were 10 times higher than the reference value. For B, only very few soil samples exceeded the reference value indicated in the same legislation (120 mg/kg [24]). Moreover, the pseudo-total concentrations of Ca, Cu, P, Mg, and Mn in the Ferragudo soils were clearly higher than those found in soils collected from a similar mining area in the Cercal-Odemira region (Rosalgar mine), where the orebody also comprised vein structures of Fe and Mn oxides, but the area is dominated by natural and autochthonous vegetation [4]. Concentrations of Cu, Mn, and Zn were also higher than those found in uncontaminated soils of the region [21]. Concentrations of Na in the sampled soils were somewhat high.

Although the high concentrations of some of these elements in the pseudo-total fraction can be associated with the mine wastes incorporated in natural soils (e.g., Mn and Cu), amounts of nutrients such as Ca, P, and Mg (Table 2) can suggest the application of amendments to promote pasture development. In fact, these concentrations, as well as the contents of exchangeable Ca and Mg, total N, and extractable P determined in the studied soils (Table 1), were much higher compared to those in uncontaminated soils from the region and not used for agricultural purposes [21].

Even if the pseudo-total concentrations of the elements in the Ferragudo soils were high (Table 2), the soil available fractions of the same elements, corresponding to the elements in soil solution and retained in the exchangeable complex of colloidal fractions, were generally low (<8.4% of the pseudo-total concentrations; Table 3). Exceptions were obtained for Ca in all soils and Na in some soil samples under oak trees (Ca 11–35% and Na 9.7–22% of pseudo-total concentrations; Table 3). The available fraction of these elements, as well as Mg, was correlated with their pseudo-total concentrations (r > 0.80, *p* < 0.05). The availability of Ca in holm oak soils also seems to be related to the organic C content (r ≈ 0.86, *p* < 0.05). For the other elements, no correlations were found between the concentrations in the soil available fraction and pH, organic C concentrations, and pseudo-total concentrations.

In soils under holm oak trees, the available fraction of K, S, and B decreased significantly with depth (Table 3), similar to elements shown in Table 2, justifying the decrease in the EC in the same pattern. However, the concentrations of Cu and Mn in the same fraction increased with depth.

Dehydrogenase activity is an important indicator of the functionality of the overall soil microbial community, as it is an intracellular enzyme present in all living organisms and organic matter decomposition [25]. The study soils showed great heterogeneity in the dehydrogenase activity, independently of plant system and depth (Table 4). In soils under holm oak trees, the enzymatic activity decreased significantly with the depth. The lowest dehydrogenase activity (0.2 µg TPF. g .16 h^−1^) was obtained in a soil sample collected between 10 and 20 cm depth under holm oak plants, which also presented the lowest N concentration (0.3 g/kg). Although soil depth may contribute to a decrease in dehydrogenase activity due to a possible decrease in aeration, other soil physicochemical characteristics may influence dehydrogenase activity [26]. In fact, the enzymatic activity in soils under holm oaks was related to two important nutrients in the soil system, total N (r ≈ 0.85, *p* < 0.01) and available S (r ≈ 0.93, *p* < 0.01), as well as organic C content (r ≈ 0.79 *p* < 0.01).

Dehydrogenase activities in soils from herbaceous root systems (Table 4) were in the same range as those in the surface layer of holm oak soils and also correlated with total N concentration in the soils (r = 0.89, *p* < 0.01).

The agrosilvopastoral system included a low density of holm oak (*Quercus ilex* L.), with good development and a continuous herbaceous cover of significant density. This herbaceous cover was mainly composed of species of the genera *Trifolium*, *Silene*, *Plantago*, *Anthemis*, *Ornithopus*, and different species of the family Poaceae. No visual differences were observed in the composition of herbaceous diversity and plant cover depending on soil characteristics. At the landscape scale, there were some small areas, randomly scattered, with autochthonous shrub species belonging mainly to the genus *Cistus*. In general, no significant changes were observed in the landscape of the mine area, which is similar to the adjacent areas and other agrosilvopastoral systems in the region.

Although the availability of elements in soil affects their concentrations in plants, other processes can occur in the soil-plant system (e.g., antagonism or synergism between elements, limitation of root uptake, water availability, and plant species [12,13]). Moreover, in modern pasture systems, the elemental composition of plants, especially macronutrients, can be more related to fertilizer application [27] than to their availability in the soil.

In the study area, independently of the concentration of the elements in the available fraction of the soil, different accumulation patterns were obtained according to the element and the plant system (Table 5). The shoots from the herbaceous pasture showed the highest concentrations of elements, except Mn, compared to the leaves of the holm oak. In fact, the accumulation of elements in shoots/leaves depends on the species, being a combined response of the uptake and translocation capacity of the plants and the concentration of elements in the available fraction of the soil [12]. Moreover, for Mn, interactions between elements at the root level and antagonistic reactions with Cu, Zn, and/or Fe can occur [13].

Concentrations of Cu, Fe, and Zn in both plant systems were considered normal or sufficient for plants in general, while most of the plant samples exceeded phytotoxic levels (400 mg Mn/kg [13]) for Mn. However, no visual signs of Mn phytotoxicity were observed (e.g., chlorosis and necrotic parts in old leaves, blackish-brown or red necrotic parts, and crying leaf tips [13]). In general, trees of the genus *Quercus* are reported to be accumulators of Mn in leaves [28], although the specific concentration depends on the species and edafoclimatic conditions. In studies carried out on holm oaks growing in natural areas and urban parks in the regions of Campania and Florence (Italy), Mn concentrations in the leaves were lower than those obtained in the present study [29,30]. The higher tolerance of plants to Mn in Ferragudo, since no visual phytotoxic signs were observed, can be related to this plant population. In fact, populations of several plant species (e.g., *Cistus* and *Lavandula* genus) growing in mining areas of the Iberian Pyrite Belt show higher concentrations of elements in plant tissues compared to those from uncontaminated areas, without any phytotoxic effect, due to efficient tolerance and antioxidant mechanisms [21,31,32,33].

Although no strong correlation between Mn concentration in the available soil fraction (or pseudo-total fraction) and plants was obtained, it is evident that the availability of this element in soil is high (Table 3) and should have some relationship with Mn plant amounts.

Concentrations of other elements (N, P, K, Mg, Cu, Fe, and Zn) in holm oak leaves were in the same range as those collected in different locations, land uses, and soils ([29,30,34] and references therein). According to the same authors, the N:P ratio in leaves, considered a useful nutritional indicator for plants, is very variable. The values calculated in the present study varied between 10 and 16, which are in the same range as those reported by the previous authors for the same species. However, these ratios may indicate N or P nutrient limitation depending on the sample (in general, N:P < 14 indicates N limitation, and N:P ratio > 16 indicates P limitation [35]).

The studied area is an agrosilvopastoral system where the soils had incorporated mine wastes, so it is important to assess the risk of consumption of herbaceous vegetation by domestic animals. According to Mineral Tolerances of Animals [36], the shoots of the herbaceous pasture growing in some of the sampled areas had concentrations of Fe (660.5 and 767.3 mg/kg) and K (21.1–24.0 g/kg) that exceeded the maximum tolerable levels (MTL) based on indices of animal health for cattle and sheep (500 mg Fe/kg and 20 g K/kg). Although the absolute values of these samples may indicate some caution, the mean values of all herbaceous pastures sampled were below (384 mg Fe/kg) or at the limit (20 g K/kg) of the MTL. Furthermore, Fe and K are elements that are considered to cause moderate adverse effects in animals when overfed for long periods of time [37]. Thus, it was considered that the health concern may also be low due to the existence of dilution factor with shoots consumed in adjacent areas and other food sources provided to the animals. In fact, the diet of cattle is not limited to herbaceous material from the study area. Being a dryland pasture, the amount of pasture produced is not sufficient at any time of the year, and farmers need to buy pasture or silage from other origins.

Moreover, it is reported by [36] that K and Fe concentrations in some forages can reach higher values (>50 g K/kg and 700–800 mg Fe/kg). Similar to the other types of plants, element concentrations in forages can vary with species and variety, growth stage, crop management practices, and seasons [12,27,38,39]. Therefore, the risk for consumption by domestic animals can be considered low. Similar results have been obtained in pastures grown on recovered mining wastes from high mountain areas [40].

## 3. Materials and Methods

### 3.1. Site Description

Ferragudo is an abandoned mine (≈38 ha) located in the Beja district (37°38′9″ north, 8°3′58″ west), which is part of the Portuguese sector of the Iberian Pyrite Belt [3]. The orebody, which forms stratiform and vein structures (small to medium dimension), consists of Fe and Mn oxides (pyrolusite) and Mn carbonates (rhodochrosite and rhodonite) and was intensively exploited in the forties and fifties of the twentieth-century, both in wells and galleries up to 40 m depth and in open pit. The exploitation of the orebody began in 1875 and ended in 1970, mainly due to the closure of the ore processing company. The Mn-oxides and carbonates produced concentrates with 50 and 38–48% Mn, respectively [41,42].

The environmental hazard impact of the Ferragudo mining area was considered intermediate (level three, where level five is considered with extreme impact) due to the small volume of mine wastes with relatively low total concentrations of potentially toxic elements, except for Mn [3]. After the closure, the interventions in the area resulting from the mining activity led to the spread of mine wastes over the topographic surface, which contributed to the alteration of pedogenesis processes in soils developed on different materials, depending on the area (e.g., mine wastes or a mixture of mine wastes, host rocks, and natural former soils), and sowing of herbaceous plants. The land use of the area included grazing and forestry [3].

In recent decades, Ferragudo mining area has been an agrosilvopastoral system extensively managed, which includes holm oak (*Quercus ilex* L.), with low density, spaced by a homogeneous and dense coverage of a biodiverse pasture composed mainly of species from families Poaceae and Asteraceae *(*Figure 1A,B). Although some spontaneous species can exist in the pasture land, most of them were sown. In very limited areas, especially with high stoniness, there are patches of autochthonous shrubs with *Cistus* and *Lavandula* species.

Soils from some areas within the study area are classified as Spolic Technosols [43]. The natural soils in the area adjacent to the mine are classified as Leptosols ([43], corresponding to “Litossolos” in the Portuguese soil classification [44]).

According to the Köppen classification, the region has a Mediterranean climate (Csa), with hot (average 23 °C) and dry summers (the driest month with an average of 3 mm rainfall) and moderately cold (average 10.8 °C) and wet winters. The annual rainfall (average 572 mm) occurs mostly in winter with an irregular pattern (Climatogical normal 1971–2000, Beja station [45]).

### 3.2. Sample Collection and Analysis

Due to the unclear definition of the boundaries of the old mining area, the present study selected two zones around the all open-pit where the contamination level may be higher. The zones were pasture around holm oaks where there was a strong influence of tree canopy and roots (hereafter referred to as holm oak area) and herbaceous pasture without tree cover where there was no influence of holm oaks (hereafter referred to as pasture area). Eight plots were selected in each zone (n = 16 plots in the total study area). In the spring, composite soil samples (composed of five subsamples; Figure 1C,D) and the corresponding plant samples were collected in each plot.

In the holm oak area, the soil sampling was conducted around the tree at two depths (0–10 cm and 10–20 cm) because of the existence of a clear visual differentiation of the layers, while in the pasture soils were sampled only from the layer up to 20 cm deep (each sampling plot of 0.25 m^2^, 0.50 × 0.50 m). According to the zone and in the same soil sampling plots, composite samples of mature holm oak leaves (n = 8, each sample composed of 30 leaves sampled at four cardinal points) and total shoot biomass of all herbaceous cover was collected. In the pasture, the dominant herbaceous genus and family were also identified.

Soil samples were homogenized, air-dried, and sieved at 2 mm for subsequent chemical analysis. Standard methods for soil (fraction < 2 mm) characterization were used: pH and electrical conductivity in water (1:2.5 m/V), pH in KCl (1:2.5 m/V), concentration of non-acid exchangeable cations (ammonium acetate extraction at pH 7 and determination with Flame Atomic Absorption Spectroscopy (AAS-F), total acidity (related to exchangeable acid cations) was determined by the standard method with ammonium acetate buffered to pH 7 and by volumetric determination with NaOH solution; extractable P and K (Egner-Riehm method), total nitrogen (Kjeldahl method) and total organic carbon (by combustion at 550 °C using an Analytik Jena Analyser). Cation exchange capacity was determined by the sum of non-acid cations and total acidity.

The pseudo-total fractions of the elements (Ca, Cu, Fe, K, Mg, Mn, Na, P, S, and Zn) in soils were determined by Inductively Coupled Plasma–Optical Emission Spectrometry (ICP-OES Thermo Scientific Mod. iCAP 7000 Series, Waltham, MA, USA) after digestion in a block digester with a two-acid mixture (HNO_3_–HCl) + H_2_O_2_ addition. The same elements were analyzed in a soil extractant solution composed of a mixture of different organic acids that simulate rhizosphere conditions [46]. These elements were considered from the fraction available to the plants. The enzymatic activity of soil dehydrogenase was determined by the method of [47].

Plant samples (herbaceous pasture shoots and oak holm leaves) were dried at 60 °C and ground to powder after washing with tap water and then with deionized water. The N concentration in the plant samples was determined by the Kjeldahl method. For the determination of other nutrients, plant samples were digested with concentrated pure HNO_3_ in a block digester and analyzed by AAS-F (Ca, K, Mg, Na, Cu, Zn, Mn, Fe) and UV–VIS spectroscopy (P; vanadomolybdate method).

### 3.3. Statistical Analysis

Data were analyzed using the statistical program SPSS v18.0 for Windows to generate descriptive statistics. Data from soils collected under holm oak trees were analyzed non-parametrically using the Kruskal–Wallis method (*p* < 0.05) to identify differences between the depths. Also, the differences between concentrations of the elements in the leaves of the holm oak and the shoots of the herbaceous cover were analyzed by the same statistical test. The Spearman correlation test was used to correlate the soil and plant characteristics (r > 0.75; *p* < 0.01 and 0.05). Quality control of the analyses was performed using analytical replicates, certified standards solutions, reference material (NCSDC73348), and blanks.

## 4. Conclusions

The agrosilvopastoral system established ≈45 years after the closure of the Ferragudo mine showed a good development of holm oaks and herbaceous pasture, with no differences compared to the landscape of the adjacent areas. The soils collected had concentrations of Cu, Mo, and Zn in the pseudo-total fraction (220–572 mg Cu/kg; 8.2–177 mg Mo/kg; 350–982 mg Zn/kg) that exceeded the reference values proposed by Portuguese legislation for agricultural use but the availability of these elements was small (<8.4% of the total, corresponding to <3.6 mg Cu/kg; <5.1 mg Zn/kg) and did not represent an environmental risk. Although these elements may be associated with the spread of mine wastes, other soil characteristics (e.g., neutral pH and concentrations of available Ca and Mg, total N, and extractable P) may be attributed to agricultural management. The biological activity of the soil microbial community, evaluated by dehydrogenase, was variable, being related to some chemical soil characteristics (e.g., total N).

The plants in the agrosilvopastoral system were well adapted to the soil characteristics and did not show any visual signs of toxicity related to contamination by potentially hazardous elements, especially for Mn and Fe, which are present in high concentrations in the soils (pseudo-total concentration of 34.5–93.2 g Fe/kg; 15.2–123.7 mg Mn/kg). Some areas had herbaceous plants whose shoots have high concentrations of Fe and K (660–767 mg Fe/kg and 2.1–2.4 g K/kg), but the risk to animals can be considered low because of the presence of other food sources in the animal feed. In general, this agrosilvopastoral system did not pose a significant environmental risk.

## Figures and Tables

**Table 1:** Physicochemical characteristics of the soils, collected under the holm oaks canopy and herbaceous pastures without tree cover in the agrosilvopastoral system established in the Ferragudo mining area after closure (minimum–maximum; mean ± SD; n = 8). For each parameter, values from holm oak samples with an asterisk indicate significant differences between depths.

	Holm Oak 0–10 cm	Holm Oak 10–20 cm	Herbaceous Pasture 0–20 cm
pH_H2O_	6.4–7.2 6.8 ± 0.3	6.2–6.9 6.6 ± 0.3	5.7–6.8 6.4 ± 0.4
pH_KCl_	5.3–6.8 5.9 ± 0.5 *	4.5–5.7 5.1 ± 0.5 *	4.7–6.0 5.5 ± 0.4
EC ^1^ (µS/cm)	109–360 211 ± 76 *	40.1–174 83.8 ± 45 *	114–221 152 ± 38
Organic C (g/kg)	28.2–89.1 45.1 ± 20.1 *	4.4–28.2 12.9 ± 7.5 *	20.4–87.9 42.4 ± 23.0
Total N (g/kg)	2.2–4.8 3.0 ± 0.9 *	0.3–2.7 1.2 ± 0.7 *	1.4–5.0 2.9 ± 1.2
Extractable K (mg/kg)	120–523 354 ± 122 *	41.5–147 101 ± 38 *	4.2–498 297 ± 195
Extractable P (mg/kg)	8.3–69.9 34.3 ± 17.4	1.2–91.7 37.0 ± 29.9	13.2–101 50.9 ± 42.6
CEC^2^ and exchangeable cations (cmol_c_/kg)			
CEC ^2^	17.5–49.0 29.2 ± 9.4	8.9–25.4 18.3 ± 6.2	14.6–30.2 24.0 ± 5.7
Ca	10.9–44.1 21.2 ± 10.5 *	3.2–15.6 10.3 ± 5.1 *	12.8–20.4 17.2 ± 3.2
Mg	3.7–8.6 6.8 ± 1.8	4.2–9.1 7.1 ± 1.9	0.3–9.5 5.7 ± 3.1
Na	0.4–0.7 0.5 ± 0.1	0.3–0.8 0.6 ± 0.2	0.2–0.7 0.4 ± 0.2
K	0.3–0.9 0.6 ± 0.2 *	0.1–0.3 0.2 ± 0.1 *	0.3–0.9 0.6 ± 0.2
Total acidity	0.1–0.2 0.1 ± 0.1	0.1–0.2 0.1 ± 0.1	0.1–0.3 0.2 ± 0.1

^1^ Electrical conductivity; ^2^ cation exchange capacity.

**Table 2:** Pseudo-total concentrations of chemical elements in soils, collected under holm oaks canopy and herbaceous pastures without tree cover in the agrosilvopastoral system established in the Ferragudo mining area after closure (minimum–maximum; mean ± SD; n = 8). For each element, values from holm oak samples with an asterisk indicate significant differences between depths.

	Elements	Holm Oak 0–10 cm	Holm Oak 10–20 cm	Herbaceous Pasture 0–20 cm
g/kg	Ca	4.2–14.3 7.1 ± 3.2 *	0.8–5.9 3.9 ± 1.8 *	3.2–7.1 5.7 ± 1.4
	K	2.0–3.8 3.0 ± 0.6 *	1.1–2.6 2.1 ± 0.5 *	1.9–3.8 3.0 ± 0.7
	Mg	4.1–6.1 5.1 ± 0.8	3.8–7.2 5.3 ± 1.4	3.5–9.2 5.2 ± 1.9
	P	0.5–1.2 0.7 ± 0.2	0.3–1.2 0.6 ± 0.3	0.5–1.3 0.8 ± 0.3
	S	0.6–0.8 0.7 ± 0.1 *	0.1–0.6 0.4 ± 0.2 *	0.6–0.8 0.7 ± 0.1
	Fe	35.0–82.9 48.5 ± 16.5	35.0–93.2 56.9 ± 20.1	34.5–80.1 47.6 ± 16.9
	Mn	27.9–115 88.4 ± 28.4	15.2–110 68.8 ± 31.3	36.7–124 86.1 ± 29.2
mg/kg	B	66.4–149 91.6 ± 28.9	59.1–157 101 ± 32.6	59.5–141 88.7 ± 29.4
	Cu	121–462 302 ± 106	124–572 315 ± 179	106–446 246 ± 116
	Mo	35.7–126 92.9 ± 27.3	8.2–127 73.3 ± 40.9	21.9–177 87.4 ± 47.7
	Na	364–1730 1207 ± 443	294–1421 861 ± 374	372–2093 1132 ± 576
	Zn	162–734 433 ± 168	189–941 497 ± 276	157–982 409 ± 266

**Table 3:** Concentrations of chemical elements in the available fraction of soils, collected under holm oaks canopy and herbaceous pastures without tree cover, in the agrosilvopastoral system established in the Ferragudo mining area after closure (minimum–maximum; mean ± SD; n = 8). For each element, values from holm oak samples with an asterisk indicate significant differences between depths.

Elements	mg/kg		
	Holm Oak 0–10 cm	Holm Oak 10–20 cm	Herbaceous Pasture 0–20 cm
Ca	455–2025 1031 ± 445 *	266–903 602 ± 256 *	730–960 840 ± 83.1
K	15.8–54.9 34.0 ± 12.8 *	8.9–46.9 18.4 ± 13.09 *	8.4–73.2 44.0 ± 22.7
Mg	268–430 370 ± 65.5	266–431 352 ± 66.5	204–478 326 ± 86.8
P	3.5–8.1 5.6 ± 1.5	2.2–14.1 5.3 ± 4.4	1.0–18.8 7.5 ± 7.2
S	12.1–19.3 16.1 ± 2.4 *	6.3–15.0 10.1 ± 3.0 *	10.1–16.7 12.4 ± 2.1
Fe	6.7–37.2 17.2 ± 11.4	7.5–136 41.6 ± 44.2	3.6–57.2 18.0 ± 18.8
Mn	25.9–554 398 ± 169 *	493–791 619 ± 103 *	297–573 442 ± 93.5
B	0.5–1.4 1.1 ± 0.3 *	0.2–1.1 0.6 ± 0.3 *	0.2–0.5 0.4 ± 0.1
Cu	0.1–0.7 0.4 ± 0.2 *	0.1–3.6 1.3 ± 1.2 *	0.1–0.6 0.3 ± 0.2
Na	64.4–142 92.6 ± 23.6	43.1–140 101 ± 34.6	30.5–150 66.8 ± 40.5
Zn	0.2–3.6 1.9 ± 1.0	0.8–5.1 3.0 ± 1.4	0.9–4.4 2.0 ± 1.3

**Table 4:** Activity of dehydrogenase in soils collected under holm oaks trees canopy and herbaceous pastures without tree cover in the agrosilvopastoral system established in the Ferragudo mining area after closure (minimum–maximum; mean ± SD; n = 8). Values from holm oak samples with an asterisk indicate significant differences between depths.

µg TPF. g .16 h^−1^		
Holm Oak 0–10 cm	Holm Oak 10–20 cm	Herbaceous Pasture 0–20 cm
29.2–172 93.8 ± 51.0 *	0.2–85.6 22.6 ± 28.7 *	36.1–216 90.4 ± 71.5

**Table 5:** Concentrations of chemical elements in oak leaves and shoots of herbaceous pasture collected in the agrosilvopastoral system established in the Ferragudo mining area after closure (minimum–maximum; mean ± SD; n = 8). Values for the same element with an asterisk indicate significant differences between plants.

	Elements	Holm Oak Leaves	Herbaceous Pasture
g/kg	Ca	3.9–5.9 4.7 ± 0.6 *	3.8–9.8 6.7 ± 1.9 *
	K	4.2–6.8 5.3 ± 0.9 *	13.5–24.0 20.0 ± 0.4 *
	Mg	1.4–1.9 1.6 ± 0.2 *	2.4–4.0 3.3 ± 0.5 *
	N	14.9–19.6 17.1 ± 1.7 *	19.9–28.4 23.4 ± 2.7 *
	P	1.0–1.5 1.3 ± 0.2 *	2.5–4.0 3.4 ± 0.6 *
mg/kg	Fe	149–251 183 ± 33 *	154–767 384 ± 222 *
	Mn	197–2273 1429 ± 677 *	197–755 469 ± 197 *
	Cu	8.2–20.5 11.7 ± 4.4	7.6–14.0 10.6 ± 2.2
	Na	78.9–272 189 ± 67.6 *	1611–7732 3892 ± 1939 *
	Zn	24.6–33.4 28.0 ± 2.8 *	35.6–55.8 43.1 ± 7.6 *

## Data Availability

Data are contained within the article.

## References

[1] Liu L.W., Li W., Song W.P., Guo M.X. (2018). Remediation techniques for heavy metal contaminated soils: Principles and applicability. Sci. Total Environ..

[2] Santos E.S., Abreu M.M., Magalhães M.C.F., Bech J., Bini C., Pashkevich M. (2017). Hazard assessment of soils and spoils from the Portuguese Iberian Pyrite Belt mining areas and their potential reclamation. Assessement, Restoration and Reclamation of Mining Influenced Soils.

[3] Matos J.X., Martins L.P. (2006). Reabilitação Ambiental de áreas mineiras do Sector Português da Faixa Piritosa Ibérica: Estado da Arte e Prespectivas Futuras. Bol. Geol. Min..

[4] Rossini-Oliva S., Santos E.S., Abreu M.M. (2019). Accumulation of Mn and Fe in aromatic plant species from abandoned Rosalgar mine and their potential human risk. Appl. Geochem..

[5] Santos Oliveira J.M., Farinha J., Matos J.X., Ávila E., Rosa C., Canto Machado M.J., Daniel E.S., Martins L., Machado Leite M.R. (2002). Diagnóstico ambiental das principais áreas mineiras degradadas do pais. Bol. Minas.

[6] Santos E.S., Abreu M.M., Macías F., de Varennes A. (2014). Improvement of chemical and biological properties of gossan mine wastes following application of amendments and growth of *Cistus ladanifer* L.. J. Geochem. Explor..

[7] Tordoff G.M., Baker A.J.M., Willis A.J. (2000). Current approaches to the revegetation and reclamation of metalliferous mine wastes. Chemosphere.

[8] Weil R.R., Brady N.C. (2017). The Nature and Properties of Soils.

[9] Venkatesh G., Gopinath K.A., Ramana D.B.V., Kumari V., Srinivas I., Shanker A.K., Rao K.V., Prasad J.V.N.S., Sammi K.R., Sridhar K.B. (2024). Agrosilvopastoral systems for improved crop and fodder productivity and soil health in the rainfed environments of South India. Agric. Syst..

[10] Moreno G., Franca A., Pinto Correia M.T., Godinho S. (2014). Multifunctionality and dynamics of silvopastoral systems. Options Méditerr..

[11] Schnabel S., Dahlgren R.A., Moreno-Marcos G. (2013). Soil and water dynamics. Mediterranean Oak Woodland Working Landscapes.

[12] Abreu M.M., Bech J., Carvalho L.C., Santos E.S., Bini C., Bech J. (2014). Potential hazardous elements fluxes from soil to plants and the food chain. Environment and Human Health: Potentially Harmful Elements in the Environment and the Impact on Human Health.

[13] Kabata-Pendias A. (2021). Trace Elements in Soils and Plants.

[14] Dutta M., Kushwaha A., Kalita S., Devi G., Bhuyan M. (2019). Assessment of bioaccumulation and detoxification of cadmium in soil-plant insect food chain. Bioresour. Technol. Rep..

[15] Kovacheva A., Vladov I., Gabrashanska M., Rabadjieva D., Tepavitcharova S., Nanev V., Dassenakis M., Karavoltsos S. (2020). Dynamics of trace metals in the system water–soil–plant–wild rats–tapeworms (*Hymenolepis diminuta*) in Maglizh area, Bulgaria. J. Trace Elem. Med. Biol..

[16] Madejón P., Domínguez M.T., Murillo J.M. (2009). Evaluation of pastures for horses grazing on soils polluted by trace elements. Ecotoxicology.

[17] Rossini-Oliva S., Nuñez R.L. (2021). Potential toxic elements accumulation in several food species grown in urban and rural gardens subjected to different conditions. Agronomy.

[18] Rossini-Oliva S., Nuñez R.L. (2024). Is it healthy urban agriculture? Human exposure to potentially toxic elements in urban gardens from Andalusia, Spain. Environ. Sci. Pollut. Res..

[19] de Varennes A. (2003). Produtividade dos Solos e Ambiente.

[20] Veloso A., Sempiterno C., Calouro F., Rebelo F., Pedra F., Castro I.V., da Conceição Gonçalves M., da Encarnação Marcelo M., Pereira P., Fareleira P. (2022). Manual de Fertilização das Culturas.

[21] Abreu M.M., Santos E.S., Ferreira M., Magalhães M.C.F. (2012). *Cistus salviifolius* a promising species for mine wastes remediation. J. Geochem. Explor..

[22] Khalil M.I., Cordovil C.M.d.S., Francaviglia R., Henry B., Klumpp K., Koncz P., Llorente M., Madari B.E., Muñoz-Rojas M., Rainer N., FAO, ITPS (2021). Mediterranean savanna-like agrosilvopastoral grassland system in Spain, Italy and Portugal. Recarbonising Global Soils—A Technical Manual of Recommended Sustainable Soil Management Volume 4: Cropland, Grassland, Integrated Systems and Farming Approaches—Case Studies.

[23] Moreno G., Bartolome J.W., Gea-Izquierdo G., Cañellas I., Campos P., Huntsinger L., Oviedo J.L., Starrs P.F., Diaz M., Standiford R.B., Montero G. (2013). Overstory-understory relationships. Mediterranean Oak Woodland Working Landscapes. Dehesas of Spain and Ranchlands of California.

[24] Agência Portuguesa do Ambiente (2019). Solo Contaminados—Guia Técnico: Valores de Referencia Para o Solo.

[25] Dick W.A., Tabatabai M.A., Metting B. (1993). Significance and potential uses of soil enzymes. Soil Microbial Ecology.

[26] Wolińska A., Stępniewska Z., Canuto R.A. (2012). Dehydrogenase activity in the soil environment. Dehydrogenases.

[27] Correia A.D. (1986). Bioquímica nos Solos, nas Pastagens e Forragens.

[28] Bargagli R. (1998). Trace Elements in Terrestrial Plants: An Ecophysiological Approach to Biomonitoring and Biorecovery.

[29] Maisto G., Baldantoni D., De Marco A., Alfani A., De Santo A.V. (2013). Ranges of nutrient concentrations in *Quercus ilex* leaves at natural and urban sites. J. Plant Nutr. Soil Sci..

[30] Ugolini F., Tognetti R., Raschi A., Bacci L. (2013). *Quercus ilex* L. as bioaccumulator for heavy metals in urban areas: Effectiveness of leaf washing with distilled water and considerations on the trees distance from traffic. Urban For. Urban Green..

[31] Arenas-Lago D., Santos E.S., Carvalho L.C., Abreu M.M., Andrade M.L. (2018). *Cistus monspeliensis* L. as a potential species for rehabilitation of soils with multielemental contamination under Mediterranean conditions. Environ. Sci. Pollut. Res..

[32] Santos E.S., Abreu M.M., Saraiva J.A. (2016). Mutielemental concentration and physiological responses of *Lavandula pedunculata* growing in soils developed on different mine wastes. Environ. Pollut..

[33] Santos E.S., Abreu M.M., Nabais C., Saraiva J.A. (2009). Trace elements and activity of antioxidative enzymes in *Cistus ladanifer* L. growing on an abandoned mine area. Ecotoxicology.

[34] Monaci F., Ancora S., Paoli L., Loppi S., Franzaring J. (2022). Differential elemental stoichiometry of two Mediterranean evergreen woody plants over a geochemically heterogeneous area. Perspect. Plant Ecol. Evol. Syst..

[35] Koerselman W., Meuleman A.F.M. (1996). The vegetation N:P ratio: A new tool to detect the nature of nutrient limitation. J. Appl. Ecol..

[36] National Research Council (2005). Mineral Tolerance of Animals.

[37] Weiss W.P. Mineral Tolerances of Animals. Proceedings of the Tri-State Dairy Nutrition Conference.

[38] Underwood E.J. (1977). Trace Elements in Human and Animal Nutrition.

[39] Underwood E.J., Suttle N.F. (1999). The Mineral Nutrition of Livestock.

[40] Santos E., Arán D. Linking circular economy and environmental rehabilitation in the designed Technosols for highmountain pastures implementation. Proceedings of the EGU General Assembly.

[41] Matos J., Rego M. (2007). Santa Bárbara de Padrões—Geologia. Santa Bárbara de Padrões—Fragmentos de Memória.

[42] Matos J., Pereira Z., Rego M. (2013). As Minas do Campo Branco, Castro Verde.

[43] IUSS Working Group WRB (2022). World Reference Base for Soil Resources. International Soil Classification System for Naming Soils and Creating Legends for Soil Maps.

[44] Direção-Geral de Agricultura e Desenvolvimento Rural Sistema Nacional de Informação de Solos. Cartografia de Solos (Sul) 1:25000. https://portalgeo.dgadr.pt/portal/apps/webappviewer/index.html?id=7cf1a919b54f4a69827f845f10bbfb8d.

[45] Instituto Português do Mar e da Atmosfera. https://www.ipma.pt/bin/file.data/climate-normal/cn_71-00_BEJA.pdf).

[46] Feng M., Shan X., Zhang S., Wen B. (2005). A comparison of rhizosphere-based method with DTPA, EDTA, CaCl_2_ and NaNO_3_ extraction methods for prediction of bioavailability of metals in soil to barley. Environ. Pollut..

[47] Tabatabai M.A., Mickelson S.H., Bigham J.M. (1994). Soil enzymes. Methods of Soil Analysis.

